# Data-driven neurobiological subtyping of Parkinson’s disease using diffusion MRI-derived isotropic diffusion

**DOI:** 10.1007/s00234-026-03939-4

**Published:** 2026-02-09

**Authors:** Anupa A Vijayakumari, Ken E Sakaie, Daniel Teixeira-Dos-Santos, Hubert H Fernandez, Benjamin L Walter

**Affiliations:** Cleveland Clinic, Cleveland, United States

**Keywords:** Machine learning, Parkinson’s disease, Diffusion MRI, Cluster analysis, Extracellular water, Isotropic diffusion

## Abstract

**Purpose:**

Parkinson’s disease (PD) exhibits marked clinical and biological heterogeneity. This study aimed to identify neurobiologically defined PD subtypes using isotropic diffusion (ISO), a diffusion MRI-derived metric sensitive to changes in isotropic water diffusion associated with microstructural alterations, and to determine whether these subtypes differ in baseline motor profiles and longitudinal change in imaging and motor scores.

**Methods:**

Baseline ISO values were extracted from 12 subcortical motor regions in 156 de novo PD patients from the Parkinson’s Progression Markers Initiative. Hierarchical clustering was applied to ISO values to derive data-driven subtypes. Baseline differences in motor severity were assessed using independent two-sample t-tests. Longitudinal change in ISO and motor outcomes over four years was evaluated using baseline-adjusted change-score regression models in patients with complete follow-up data (*n* = 78).

**Results:**

Two subtypes emerged: subtype 1 (*n* = 62) with lower ISO values and subtype 2 (*n* = 94) with higher ISO across all regions. Subtype 2 showed greater baseline rigidity and bradykinesia. In longitudinal analyses (subtype 1, *n* = 34; subtype 2, *n* = 44), no significant differences were observed between subtypes in change in ISO across subcortical regions or in progression of motor scores over four years.

**Conclusion:**

Our findings indicate that ISO-derived subtypes are associated with distinct baseline neurobiological and motor profiles in early PD, but do not show evidence of differential motor or imaging progression over a four-year follow-up. This pattern highlights the complexity of PD heterogeneity and underscores the need for further investigation in larger, long-term cohorts.

**Supplementary Information:**

The online version contains supplementary material available at 10.1007/s00234-026-03939-4.

## Introduction

Parkinson’s disease (PD) is a progressive neurodegenerative disorder characterized by the selective loss of dopaminergic neurons in the substantia nigra and the accumulation of α-synuclein pathology throughout the brain [1, 2]. While PD is traditionally diagnosed based on cardinal motor symptoms including bradykinesia, rigidity, tremor, and postural instability and gait difficulty (PIGD), clinical presentation and disease progression vary considerably among individuals [3, 4]. This heterogeneity has significant implications for prognosis, treatment response, and clinical trial design, underscoring the need for biological markers that can better define PD subtypes.

Diffusion MRI provides a powerful tool to investigate microstructural changes associated with PD [5]. Prior work using bi-compartment diffusion models has shown that extracellular water–sensitive metrics, such as free water, are elevated in basal ganglia and motor circuits, reflecting neuroinflammatory and neurodegenerative processes [6–10]. Notably, Bower et al. demonstrated that patients classified clinically as PIGD exhibited greater free-water increases compared to tremor-dominant patients [10]. While this finding highlights the biological sensitivity of diffusion-derived metrics, the subtypes were defined a priori by clinical phenotype rather than emerging directly from imaging data. Thus, the identification of subtypes grounded directly in neurobiological markers, including those sensitive to extracellular pathology, remains an unmet need.

Isotropic diffusion (ISO), a model-free diffusion metric that is derived within the generalized Q-sampling Imaging (GQI) framework [11], quantifies the isotropic component of water diffusion arising from cerebrospinal fluid, edema, or tissue loss. Higher ISO values have been linked to demyelination and edema [12], suggesting that ISO can capture disease-related alterations. Yet, whether ISO can stratify PD into neurobiologically distinct subgroups that exhibit different trajectories of neurodegenerative brain changes as well as motor progression has not been investigated.

To address this gap, the present study aimed to apply unsupervised clustering to baseline ISO values extracted from subcortical motor regions in a cohort of de novo PD patients from the Parkinson’s Progression Markers Initiative (PPMI) [13, 14]. Our objectives were to (1) identify distinct PD subtypes based on baseline ISO patterns, (2) characterize their baseline motor phenotypes, and (3) examine whether these ISO-defined subtypes differ in longitudinal change in ISO measures and motor severity over a 4-year follow-up.

## Methods

### Participants

We included 156 de novo Parkinson’s disease (PD) patients from the PPMI database (https://www.ppmi-info.org/access-data-specimens/download-data; RRID: SCR_006431), accessed through the standard application process. Inclusion criteria were: diagnosis of PD within the past two years; a positive DaTscan confirming diagnosis; no dopaminergic treatment within six months of enrollment; availability of diffusion MRI data at baseline and at the 4-year follow-up; and MDS-UPDRS-III motor scores obtained at baseline (in the drug-naïve state) and at the 4-year follow-up (in the OFF-medication state). Patients were excluded if they had dementia or atypical parkinsonian syndromes, significant neurological or psychiatric conditions, or with structural brain abnormalities, poor-quality imaging data (e.g., motion artifacts, or susceptibility distortions). At the 4-year follow-up, only 78 patients had both diffusion MRI data and complete OFF-medication MDS-UPDRS-III assessments due to attrition and incomplete follow-up data.

### Ethics approval

The PPMI study was conducted in accordance with the Declaration of Helsinki and Good Clinical Practice guidelines and is registered on ClinicalTrials.gov (NCT01141023). All participants provided written informed consent under protocols approved by the local ethics committees of participating sites, listed at https://www.ppmi-info.org/about-ppmi/ppmi-clinical-sites. One of the authors (AAV) obtained permission to access tier 3 PPMI data, which were provided in a fully de-identified format. The PPMI Data and Publications Committee reviewed and administratively approved this manuscript in accordance with PPMI data use policies. As all data were de-identified and previously collected under approved protocols, no additional ethics committee approvals were required for this analysis.

### Image acquisition

Diffusion MRI data was acquired using a Siemens 3 T TrioTim MRI scanner equipped with a 12-channel Matrix head coil. Data from baseline and 4-year timepoints were downloaded for analysis. The diffusion sequence employed a two-dimensional echo-planar imaging (EPI) protocol with the following acquisition parameters: repetition time (TR) = 900 ms; echo time (TE) = 88 ms; flip angle = 90°; voxel dimensions = 2 × 2 × 2 mm³; 72 axial slices; and 64 diffusion-encoding directions with a b-value of 1000 s/mm². Additionally, a single non-diffusion-weighted (b = 0 s/mm²) volume was included. Further details of the PPMI study design and imaging protocols are available in Marek et al. [14] and the PPMI MRI Operations Manual [https://www.ppmi-info.org/wp content/uploads/2017/06/PPMI-MRI-Operations-Manual-V7.pdf].

### Image processing

Diffusion MRI data were processed using DSI Studio (http://dsi-studio.labsolver.org; version 2024 release), a widely used software platform for diffusion model reconstruction and extraction of microstructural metrics [15, 16]. Preprocessing included correction for motion and eddy current distortions to enhance data quality and mitigate artifacts. Raw images were converted into DSI Studio’s native.src format. Reconstruction was performed using GQI, a model-free framework that estimates spin distribution functions (SDFs) and nonlinearly registers them to the standard ICBM-152 space [17]. Reconstruction quality was assessed using the goodness-of-fit coefficient (R²) between each subject’s anisotropy map and the standard space [18]. Only datasets meeting a threshold of R² > 0.70 were included. Each reconstructed dataset was saved as a.fib file, which encodes voxel-wise diffusion metrics, including the isotropic diffusion (ISO) value. The ISO metric used here is model-free and derived from GQI, potentially offering increased robustness to partial volume effects in small subcortical structures [11, 19]. ISO quantifies the direction-independent (isotropic) component of the diffusion signal, serving as a proxy for extracellular water and capturing diffusion related to cerebrospinal fluid (CSF), edema, or neurodegenerative tissue loss.

We extracted ISO values using the connectometry module in DSI Studio [20] for twelve PD–relevant subcortical brain regions of interest (ROIs) defined by the ATAG atlas [21], including bilateral red nucleus (RN), substantia nigra (SN), subthalamic nucleus (STN), striatum, globus pallidus externus (GPe) and internus (GPi). These subcortical regions were selected because of their involvement in PD pathologic changes that occur early in the disease [22–25]. For each subject and time point (baseline and 4-year), mean ISO values were computed for each ROI. These regional ISO metrics were used in subsequent clustering and statistical analyses.

### Motor outcome measures

Motor symptoms were assessed using Part III of the Movement Disorder Society-sponsored revision of the Unified Parkinson’s Disease Rating Scale (MDS-UPDRS-III) [26]. Total scores and subdomain scores were extracted, including rigidity (item 3.3), bradykinesia (items 3.4–3.8), postural instability and gait difficulty (PIGD; items 3.10–3.13), and tremor (items 3.15–3.17), at two time points: baseline and 4 years. To minimize the influence of dopaminergic medication and capture disease-related motor progression, only OFF-medication scores were included. After filtering for OFF-state availability, 78 patients had usable motor data at the 4-year visit.

### Data-driven clustering

**Fig. 1:**
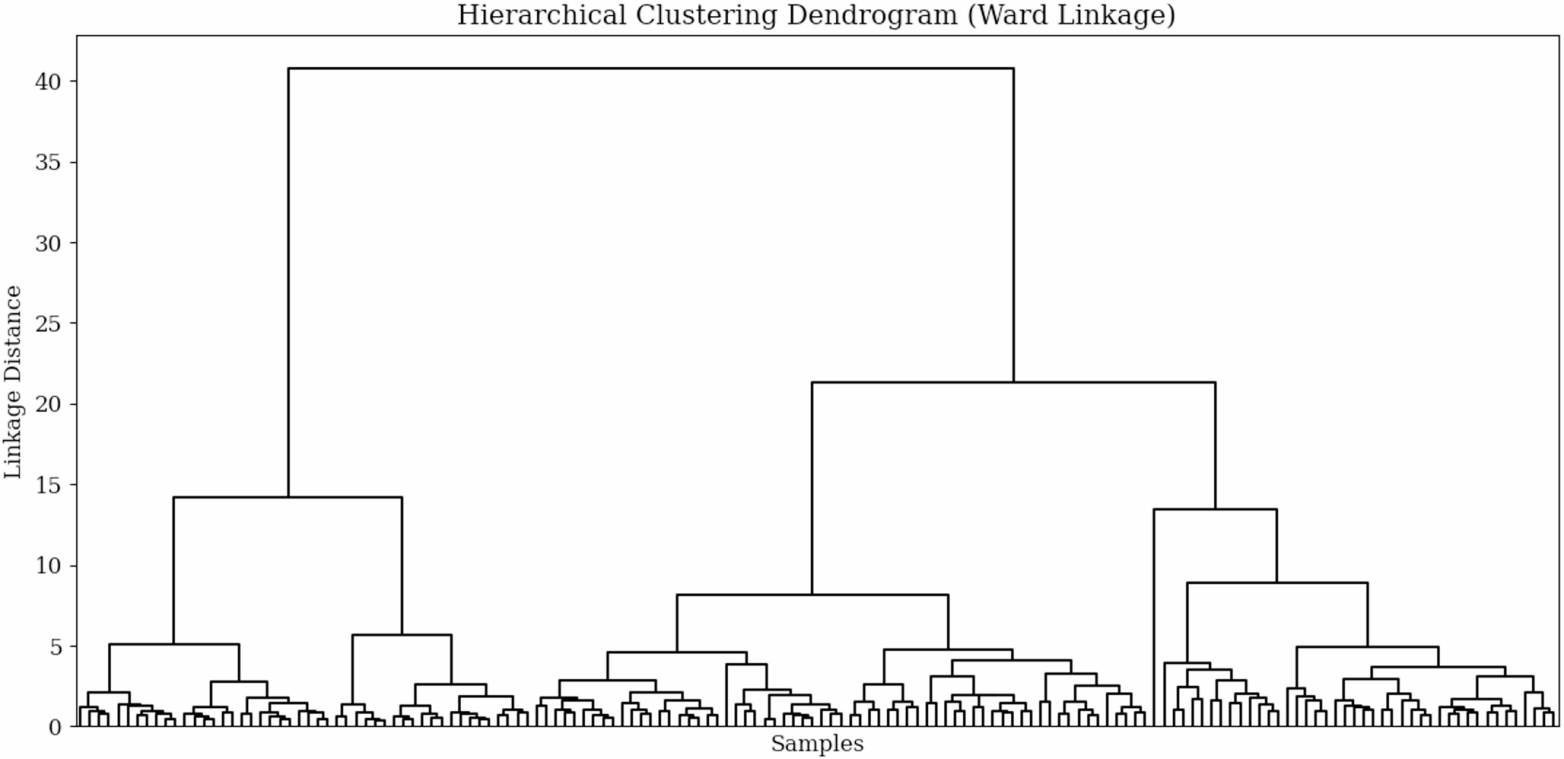
Hierarchical clustering dendrogram based on baseline isotropic diffusion (ISO) values. ISO values from 12 PD-relevant subcortical brain regions (bilateral red nucleus, substantia nigra, subthalamic nucleus, striatum, globus pallidus externus, and globus pallidus internus) were used as input features

Hierarchical agglomerative clustering was applied to identify subtypes based on ISO values extracted from 12 PD-relevant brain regions (bilateral SN, STN, RN, striatum, GPi and GPe). No clinical variables were used in the clustering. All ISO features were z-score standardized across participants to ensure comparability. Clustering was performed using Ward’s linkage and Euclidean distance, implemented via the scipy.cluster.hierarchy library in Python. A dendrogram was generated to visualize hierarchical distances (Fig. 1).

To determine the optimal number of clusters, the Calinski–Harabasz (C-H) index was computed for clustering solutions ranging from k = 2 to 10. The C-H index evaluates the ratio of between-cluster dispersion to within-cluster dispersion, with higher values indicating more distinct clustering structure. We also assessed cluster stability using bootstrap resampling with 100 iterations, in which hierarchical clustering was repeated on resampled datasets and agreement with the original clustering was quantified using the adjusted Rand index (ARI). It is a measure of similarity between two cluster labelings that corrects for chance agreement (range: −1 to 1), with higher values indicating stronger agreement [27]. Based on these validation metrics, a two-cluster solution was selected (see Results), and final cluster assignments were derived using the fcluster() function with maxclust = 2.

### Statistical analysis

Baseline group differences in demographic, clinical, and ISO variables were assessed. Independent two-sample t-tests were used to compare ISO values between subtypes across 12 PD-relevant brain regions defined by the ATAG atlas, thereby verifying that the clustering procedure produced neurobiologically distinct groups. Age differences were evaluated using independent two-sample t-tests, while sex distribution was compared using chi-square tests. We also determined whether these ISO-based subtypes differed in motor severity at baseline using independent two-sample t-tests. Effect sizes were quantified using Cohen’s *d* to facilitate interpretation of the magnitude of subtype differences.

For patients with complete baseline and 4-year data (*n* = 78), longitudinal changes in motor severity and ISO measures were assessed using a baseline-adjusted change-score framework. Baseline adjustment was included to control for initial group differences, mitigate regression-to-the-mean effects and isolate the effect of subtype on rate of change. Change scores were calculated as the difference between the 4-year and baseline assessments (Δ = follow-up − baseline). For motor outcomes, changes in MDS-UPDRS Part III total score and subdomain scores were assessed using linear regression, with ISO-defined subtype as the primary predictor. Baseline motor scores for the corresponding outcome were included as a covariate, along with age and sex. Similarly, baseline-adjusted change-score analyses were performed for ISO values extracted from 12 subcortical regions of interest. For each region, change in ISO was modeled as the dependent variable, with ISO-defined subtype as the primary predictor and baseline ISO from the same region, age, and sex included as covariates.

To correct for multiple comparisons, false discovery rate (FDR) correction was applied using the Benjamini-Hochberg procedure (*p* < 0.05). All statistical analyses were conducted using Python (v3.11.13), with core packages including pandas, scipy, scikit-learn, statsmodels, and matplotlib.

## Results

### Baseline demographic characteristics

Subtype 1 included 62 patients (mean age 60.7 ± 9.6 years; 39 males and 23 females), and subtype 2 included 94 patients (mean age 61.0 ± 9.8 years; 61 males and 33 females). Age did not differ significantly between subtypes (*t* = −0.17, *p* = 0.87). Sex distribution was also not significantly different (*χ²* = 0.01, *p* = 0.93), indicating that ISO-based clustering was not confounded by demographic variables.

### ISO-Based clustering identified two distinct PD subtypes

Hierarchical clustering of baseline ISO values from 12 PD-relevant subcortical brain regions, extracted using the ATAG atlas, revealed two distinct subtypes (Fig. 1). The C-H index was maximized at k = 2 (C-H = 176.02; Supplementary Fig. 1), indicating that this solution provided the most favorable balance between between-cluster separation and within-cluster compactness. Bootstrap resampling analysis demonstrated good cluster stability, with a mean ARI of 0.60 across 100 iterations, indicating that the two-cluster structure was consistently recovered across resampled datasets.

At baseline, subtype 1 exhibited lower ISO levels across all regions, while subtype 2 showed elevated ISO values (all *p* < 0.05, FDR-corrected). These differences were accompanied by large effect sizes across regions (Cohen’s *d* range: −1.36 to −2.00), indicating that the clustering produced neurobiologically distinct groups (Table 1).

**Table 1:** Baseline isotropic levels across ATAG regions in ISO-Defined parkinson’s disease subtypes

ATAG regions	Subtype 1 (*n* = 62) (mean ± SD)	Subtype 2 (*n* = 94) (mean ± SD)	t	*p*	Cohen’s d
RN left	0.31 ± 0.07	0.47 ± 0.10	−10.18	< 0.001	−1.67
RN right	0.27 ± 0.09	0.42 ± 0.09	−9.86	< 0.001	−1.61
SN left	0.26 ± 0.08	0.41 ± 0.08	−10.93	< 0.001	−1.79
SN right	0.21 ± 0.07	0.32 ± 0.06	−10.67	< 0.001	−1.75
STN left	0.38 ± 0.13	0.60 ± 0.13	−9.59	< 0.001	−1.57
STN right	0.26 ± 0.10	0.41 ± 0.10	−8.31	< 0.001	−1.36
Striatum left	0.42 ± 0.11	0.64 ± 0.14	−10.48	< 0.001	−1.72
Striatum right	0.43 ± 0.11	0.66 ± 0.14	−10.75	< 0.001	−1.76
GPe left	0.22 ± 0.06	0.35 ± 0.06	−12.01	< 0.001	−1.97
GPe right	0.22 ± 0.06	0.35 ± 0.06	−11.93	< 0.001	−1.95
GPi left	0.25 ± 0.07	0.39 ± 0.07	−11.95	< 0.001	−1.96
GPi right	0.25 ± 0.07	0.39 ± 0.07	−12.22	< 0.001	−2.00

*SD* standard deviation, *ATAG* Atlas of the Basal Ganglia, *RN* Red Nucleus, *SN* Substantia Nigra, *STN* Subthalamic Nucleus, *GPe* Globus Pallidus Externa, *GPi* Globus Pallidus Interna, *t* t-statistic from independent samples t-test comparing Subtype 1 and Subtype 2. *p* p-value; all comparisons are significant after correction for multiple comparisons (*p* < 0.05, FDR corrected). Cohen’s *d*: standardized effect size (negative values indicate higher ISO in subtype 2).

### Motor severity differences at baseline

At baseline, subtype 2 exhibited significantly greater rigidity (*p* = 0.004; Cohen’s *d* = −0.48) and bradykinesia (*p* = 0.01; Cohen’s *d* = −0.39) compared to subtype 1, corresponding to small-to-moderate effect sizes. No significant differences were observed in baseline total UPDRS-III (*p* = 0.05; Cohen’s *d* = −0.32), PIGD (*p* = 0.54; Cohen’s *d* = 0.10), or tremor (*p* = 0.34; *d* = 0.15) scores, with effect sizes indicating minimal group differences (Table 2).

**Table 2:** MDS-UPDRS-III total and subdomain at baseline by ISO-defined PD subtypes

Motor scores	Subtype 1 (*n* = 62) (mean ± SD)	Subtype 2 (*n* = 94) (mean ± SD)	t	*p*	Cohen’s d
Total	18.71 ± 8.69	21.59 ± 9.14	−1.95	0.05	−0.32
Rigidity	3.52 ± 2.35	4.83 ± 2.96	−2.93	**0.004**	−0.48
Bradykinesia	7.34 ± 4.36	9.24 ± 5.17	−2.39	**0.01**	−0.39
PIGD	0.73 ± 0.91	0.65 ± 0.68	0.60	0.54	0.10
Tremor	2.84 ± 2.51	2.49 ± 2.07	0.94	0.34	0.15

*SD* standard deviation, *PIGD* Postural Instability and Gait Difficulty, *t* t-statistic from independent samples t-test comparing Subtype 1 and Subtype 2. *p* p-value; values < 0.05 indicate statistical significance. Cohen’s *d*: standardized effect size (negative values indicate greater motor severity in subtype 2).

### Longitudinal change in ISO and motor measures

Longitudinal change-score analyses were performed in 78 patients (subtype 1, *n* = 34; subtype 2, *n* = 44). ISO-defined subtypes did not differ in longitudinal change in ISO across any examined subcortical region (all *p* > 0.05; Table 3). Similarly, no subtype differences were observed in longitudinal change in MDS-UPDRS Part III total score over 4 years or in any motor subdomain, including rigidity, bradykinesia, PIGD, or tremor (all *p* > 0.05; Table 4).

**Table 3:** Baseline-adjusted longitudinal change in ISO across ATAG brain regions

ATAG regions	β	CI		*p*
		low	high	
RN left	−0.03	−0.09	0.03	0.41
RN right	−0.03	−0.07	0.02	0.41
SN left	−0.06	−0.12	0.00	0.39
SN right	−0.06	−0.14	0.01	0.39
STN left	−0.03	−0.09	0.03	0.41
STN right	−0.03	−0.08	0.03	0.42
Striatum left	−0.02	−0.09	0.05	0.60
Striatum right	−0.01	−0.08	0.06	0.71
GPe left	−0.04	−0.08	0.01	0.39
GPe right	−0.03	−0.07	0.02	0.41
GPi left	−0.03	−0.08	0.01	0.39
GPi right	−0.03	−0.08	0.01	0.39

*β* represents the subtype effect from baseline-adjusted change-score regression models (Δ = 4-year follow-up - baseline), adjusted for baseline ISO from the same region, age, and sex. *CI* indicates the 95% confidence interval. *RN* red nucleus, *SN* substantia nigra, *STN* subthalamic nucleus, *GPe* globus pallidus externa, *GPi* globus pallidus interna.

**Table 4:** Baseline-adjusted longitudinal change in MDS-UPDRS part III total and subdomain scores

Motor scores	β	CI		*p*
		low	high	
Total	0.43	−5.49	6.36	0.88
Rigidity	−0.60	−2.23	1.03	0.47
Bradykinesia	0.56	−2.28	3.41	0.69
PIGD	0.28	−0.50	1.07	0.47
Tremor	0.33	−0.92	1.57	0.60

*β* represents the subtype effect from baseline-adjusted change-score regression models (Δ = 4-year follow-up − baseline), adjusted for baseline motor severity, age, and sex. *CI* indicates the 95% confidence interval; p values are two-sided. *MDS-UPDRS-III*: Movement Disorder Society–Unified Parkinson’s Disease Rating Scale, Part III. *PIGD*: postural instability and gait difficulty.

## Discussion

In this study, we applied a data-driven clustering approach to baseline ISO values derived from diffusion MRI to identify neurobiologically defined subtypes of PD. Two distinct subtypes emerged: one with elevated ISO across subcortical motor regions at baseline (subtype 2) and another with relatively lower ISO levels (subtype 1). These groups differed not only in baseline imaging profiles but also in motor severity, with subtype 2 exhibiting greater rigidity and bradykinesia. Although the subtypes differed neurobiologically at baseline, baseline-adjusted longitudinal analyses did not reveal significant differences in ISO or motor change over four years between subtypes.

Shippey et al. highlighted that extracellular processes play a central role in PD pathogenesis, with extracellular vesicles serving as vehicles for the spread of misfolded α-synuclein aggregates that disrupt mitochondrial function, impair axonal transport, and ultimately drive neuronal death [28]. ISO provides an in vivo marker of these extracellular processes, as it quantifies the direction-independent component of water diffusion [19]. Elevated ISO has been linked to extracellular water burden arising from microstructural alterations such as demyelination, astrogliosis, or neurodegenerative tissue loss [12]. Importantly, ISO is not specific to a single pathological mechanism but reflects the net effect of these processes. In the present study, subtype 2 patients exhibited significantly higher baseline ISO values across subcortical regions compared with subtype 1, indicating a greater baseline extracellular water burden. However, longitudinal analyses did not reveal differential ISO progression between subtypes over the four-year follow-up period. These findings suggest that extracellular pathology is a fundamental component of PD, with variability in baseline burden across patients. However, there was no clear evidence for subtype-specific differences in longitudinal change. Caution is warranted in interpreting ISO-defined subtypes, as ISO reflects isotropic water diffusion within or between cells and is influenced by multiple pathological processes, including demyelination, extracellular space expansion, edema, and neurodegenerative tissue loss [11, 15]. ISO measurements may be affected by partial volume effects, particularly in small subcortical structures where CSF contamination from adjacent ventricles can artificially inflate diffusion estimates. Such partial volume averaging can be exacerbated by atrophy. Future studies integrating imaging with histopathological data will be essential to disentangle the underlying contributors to ISO changes.

It is also important to note that another diffusion-derived metric, free water, has been widely investigated as an indicator of extracellular pathology in PD. It is derived from a bi-compartment model that separates the isotropic contribution of extracellular water from the tissue signal [29]. In PD, free water is consistently elevated across subcortical regions, and longitudinal studies indicate that it increases with disease progression and predicts decline in motor function [8–10, 30, 31]. By contrast, our study applied ISO, an alternative approach to quantifying isotropic diffusion. While ISO and free water are sensitive to extracellular water, they are derived differently and should be viewed as methodologically distinct approaches. Future work comparing ISO- and free water-defined subtypes will be needed to clarify their ability in distinguishing PD subtypes and to establish their clinical relevance.

In addition to the imaging differences, we also observed motor distinctions between ISO-defined subtypes. At baseline, subtype 2 patients exhibited worse rigidity and bradykinesia, whereas tremor and PIGD scores were comparable across groups. These findings suggest that elevated ISO burden may align with greater motor severity early in the disease course. However, longitudinal analyses did not identify significant subtype differences in change in total or domain-specific MDS-UPDRS-III scores over four years. This indicates that baseline neurobiological differences captured by ISO-based subtyping were not associated with differential motor progression over four years. It should be noted that attrition reduced the available sample for 4-year OFF-medication data to 78 patients (subtype 1, *n* = 34; subtype 2, *n* = 44), which may have limited statistical power to detect subtle group differences. Thus, caution is warranted in interpreting the longitudinal findings, and future studies with larger cohorts, longer follow-up, and more sensitive motor measures will be needed to clarify the clinical significance of neurobiologically defined subtypes.

Our findings highlight a key distinction between cross-sectional and longitudinal perspectives on PD heterogeneity. At baseline, ISO-defined subtypes demonstrated both neurobiological and clinical differences, with subtype 2 showing greater isotropic diffusion burden alongside more severe rigidity and bradykinesia. These results suggest that neurobiological subtyping can capture meaningful clinical variation early in the disease course, supporting its utility in cross-sectional settings. However, longitudinal analyses revealed that neither longitudinal changes in ISO nor motor progression differed significantly between subtypes. This dissociation emphasizes that while neurobiological markers can stratify patients at baseline, their long-term clinical impact may not be straightforward. Future studies should therefore account for both cross-sectional and longitudinal dimensions when evaluating the clinical relevance of biologically defined subtypes, as reliance on baseline differences alone may overestimate their prognostic utility.

Several limitations should be acknowledged. First, the striatum was analyzed as a single region of interest based on the ATAG atlas definition, which may reduce sensitivity to subregional striatal effects known to occur in PD, particularly within the caudate and putamen. Future studies using finer striatal parcellations may further improve the spatial specificity of ISO-based subtype characterization. Second, the clinical measures analyzed were restricted to MDS-UPDRS-III in the OFF state, which is subjective and prone to inter-subject variability [32, 33], and the use of more objective motor assessments will be important in future work [34–36]. Third, beyond motor symptoms, PD is associated with a wide range of non-motor manifestations, including cognitive impairment, rapid eye movement sleep behavior disorder, and autonomic dysfunction [37]. The present study focused on ATAG-defined subcortical regions primarily involved in motor circuits [23, 38] and did not assess whether ISO-based subtyping captures heterogeneity in non-motor symptoms. Brain regions beyond these subcortical structures, including cortical, limbic, and cerebellar circuits, are known to contribute to PD symptoms [39, 40]. Future studies incorporating non-motor outcomes and extending analyses to these regions may provide a more comprehensive characterization of PD heterogeneity. Finally, the ISO-based subtypes were not compared with established clinical subtypes or other biological markers such as CSF, free water, or molecular imaging [41–44]. Accordingly, these subtypes should be interpreted as exploratory neurobiological stratifications rather than definitive PD subtype classifications.

## Conclusion

This study shows that diffusion MRI-derived ISO identified two neurobiologically distinct subtypes of PD at baseline, characterized by higher versus lower isotropic diffusion across subcortical motor regions. However, longitudinal analyses did not reveal significant differences between subtypes in changes in ISO or motor progression over four years. Together, these findings suggest that ISO is sensitive to baseline neurobiological heterogeneity in early PD, while its ability to predict subsequent disease progression remains uncertain. Larger studies with longer follow-up and broader clinical characterization will be required to determine the prognostic and clinical relevance of ISO-based subtyping.

## Supplementary Information

Below is the link to the electronic supplementary material.

MOESM 1 (PDF 109 KB)

## Data Availability

Data were obtained from the Parkinson’s Progression Markers Initiative (PPMI; [https://www.ppmi-info.org](https://www.ppmi-info.org)) and are available to qualified investigators upon application per PPMI policies.

## Code availability

Analysis code available on reasonable request.
