# Supplementary material for: Data-driven neurobiological subtyping of Parkinson’s disease using diffusion MRI-derived isotropic diffusion
Source: Neuroradiology. 2026 Feb 9;68(7):1907–14. doi: 10.1007/s00234-026-03939-4 (PMC13407572; doi:10.1007/s00234-026-03939-4)

Supplementary Figure 1. Calinski–Harabasz (C-H) index values computed for hierarchical clustering solutions with k = 2–10 clusters. Higher values indicate greater between-cluster separation relative to within-cluster dispersion.

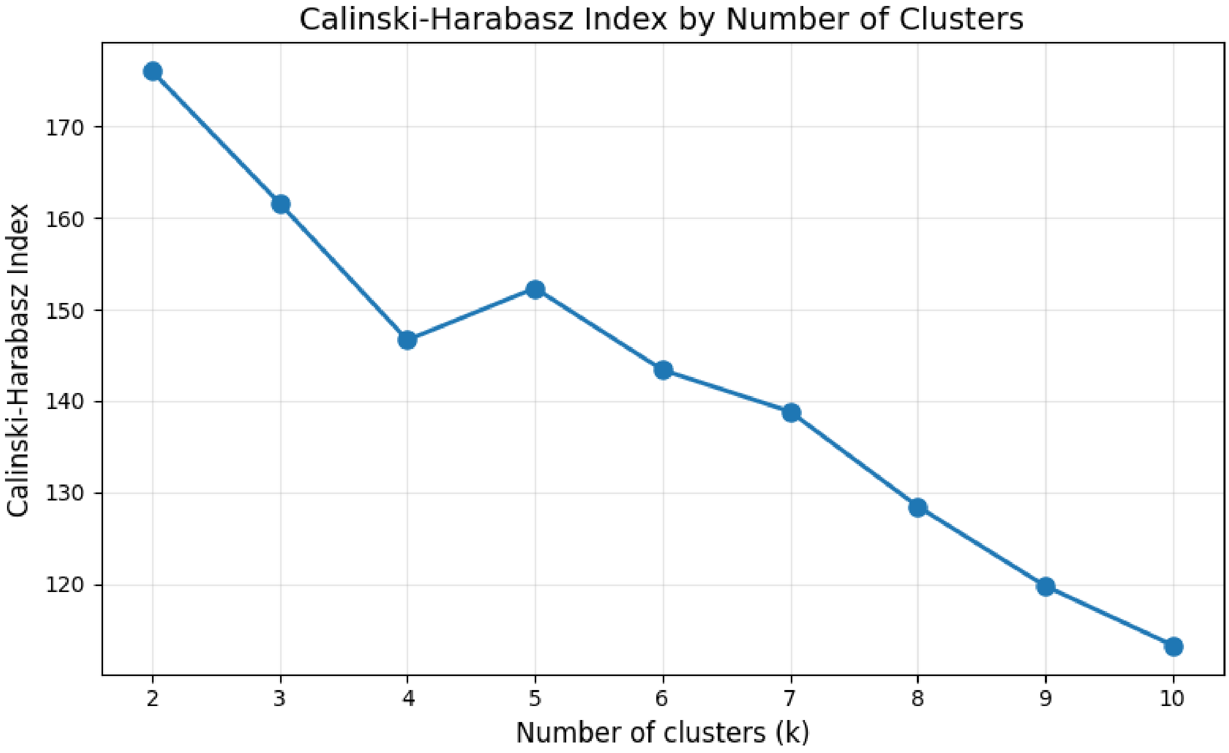

Supplement: Supplementary file 1 — (PDF 109 KB) [file 234_2026_3939_MOESM1_ESM.pdf]
